# Dystonia-deafness syndrome caused by *ACTB* p.Arg183Trp heterozygosity shows striatal dopaminergic dysfunction and response to pallidal stimulation

**DOI:** 10.1186/s11689-018-9235-z

**Published:** 2018-05-22

**Authors:** Inger Marie Skogseid, Oddveig Røsby, Ane Konglund, James P. Connelly, Bård Nedregaard, Greg Eigner Jablonski, Nadja Kvernmo, Asbjørg Stray-Pedersen, Joel C. Glover

**Affiliations:** 1 Department of Neurology, Division of Clinical Neuroscience, Oslo University Hospital, Po.box. 4950, Nydalen, 0424 Oslo, Norway; 2 Department of Medical Genetics, Division of Laboratory Medicine, Oslo University Hospital, Oslo, Norway; 3 Department of Neurosurgery, Division of Clinical Neuroscience, Oslo University Hospital, Oslo, Norway; 4 Department of Nuclear Medicine, Division of Radiology & Nuclear Medicine, Oslo University Hospital, Oslo, Norway; 5 Department of Radiology, Division of Radiology & Nuclear Medicine, Oslo University Hospital, Oslo, Norway; 6 Department of Otorhinolaryngology, Division of Head, Neck & Reconstructive Surgery, Oslo University Hospital, Oslo, Norway; 7 Institute of Clinical Medicine, University of Oslo, Oslo, Norway; 8 Baylor-Hopkins Center for Mendelian Genomics, Baylor College of Medicine, Houston, TX 77030 USA; 9 Norwegian National Unit for Newborn Screening, Division of Pediatric and Adolescent Medicine, Oslo University Hospital, Oslo, Norway; 10 Department of Molecular Medicine, Institute of Basic Medical Sciences, University of Oslo, Oslo, Norway; 11 Norwegian Center for Stem Cell Research, Department of Immunology and Transfusion Medicine, Oslo University Hospital, Oslo, Norway

**Keywords:** Dystonia-deafness syndrome, *ACTB* p.Arg183Trp, Pallidal deep brain stimulation, Striatal neuronal dysfunction, Dopaminergic dysfunction

## Abstract

**Background:**

Dystonia-deafness syndrome is a well-known clinical entity, with sensorineural deafness typically manifesting earlier than dystonia. *ACTB* p.Arg183Trp heterozygosity has been reported in six patients to cause combined infant-onset deafness and dystonia manifesting in adolescence or young adulthood. Three of these have received beneficial pallidal stimulation. Brain imaging to assess striatal function has not been reported previously, however. Nor has a comprehensive hypothesis been presented for how the pleiotropic manifestations of this specific beta-actin gene mutation originate developmentally.

**Case presentation:**

A 19-year-old girl with congenital mild dysmorphic facial features, cochlear implants for infant-onset deafness, and mild cognitive and emotional disability, presented with an adolescent-onset, severe generalized dystonia. Brain MRI and multiple single gene sequencing were inconclusive. Due to life-threatening dystonia, we implanted a neurostimulation device, targeting the postero-ventral internal pallidum bilaterally. The Burke-Fahn-Marsden Dystonia Rating Scale motor/disability scores improved from 87/25 to 21/13 at 2.5 months postoperatively, 26/14 at 3 years, and 30/14 at 4 years. Subsequent whole exome sequencing identified heterozygosity for the *ACTB* p.Arg183Trp variant. Brain imaging included ^123^I-ioflupane single photon emission computed tomography (Dopamine Transporter-SPECT), SPECT with ^123^I-epidepride (binds to dopamine type 2-receptors) and ^18^ Fluoro-Deoxy-Glucose (FDG)–PET. Both Epidepride-SPECT and FDG-PET showed reduced tracer uptake in the striatum bilaterally, particularly in the putamen. DaT-SPECT was slightly abnormal.

**Conclusions:**

In this patient with dystonia-deafness syndrome caused by *ACTB* p.Arg183Trp heterozygosity, unprecedented brain imaging findings strongly indicate striatal neuronal/dopaminergic dysfunction as the underlying cause of the dystonia. Pallidal stimulation provided a substantial improvement of the severe generalized dystonia, which is largely sustained at 4-year follow-up, and we advise this treatment to be considered in such patients. We hypothesize that the pleiotropic manifestations of the dystonia-deafness syndrome caused by this mutation derive from diverse developmental functions of beta-actin in neural crest migration and proliferation (facial dysmorphogenesis), hair cell stereocilia function (infant-onset deafness), and altered synaptic activity patterns associated with pubertal changes in striatal function (adolescent-onset dystonia). The temporal differences in developmental onset are likely due to varying degrees of susceptibility and of compensatory upregulation of other actin variants in the affected structures.

## Background

Dystonia is a movement disorder characterized by sustained or intermittent muscle contractions, causing abnormal movements, postures, or both [1] and can result from lesions or perturbed function in motor networks involving the basal ganglia, cerebellum, thalamus, and sensorimotor cortex [2]. Childhood- or adolescent-onset dystonia is often caused by acquired or genetic disorders [3], which may affect the motor networks by biochemical deficits, metabolic derangement with or without neuronal degeneration, or perturbed structural development.

Dystonia-deafness syndrome (DDS) is a well-known clinical entity, with sensorineural deafness typically manifesting earlier than dystonia. The underlying etiology varies and is often difficult to pinpoint. In a recent series of 20 patients, the cause was genetic in five, acquired in two, and unknown in 13 [4].

We present a female who in childhood received cochlear implants for infant-onset sensorineural deafness, exhibited mild dysmorphic facial features, finger contractures, constipation, and stagnation of psychosocial skills. During adolescence, she developed generalized dystonia, which at age 19 had become life-threatening and was treated with deep brain stimulation (DBS) of the internal pallidum bilaterally. At age 22, whole exome sequencing identified *ACTB* p.Arg183Trp heterozygosity, first described in twin brothers with DDS [5, 6]. Four similar patients were recently reported [7–9], and the variant associated with the Baraitser-Winter cerebrofronto-facial syndrome (BWCFF type 1 (BRWS1, OMIM#243310) [10].

The main aim of this case report is to present 4-year follow-up indicating the marked, life-improving benefits of pallidal stimulation, and brain imaging findings that strongly indicate loss of normal function of striatal neurons, including those harboring dopamine type 2 receptors, as the cause of the dystonia. We also propose a hypothesis for the pleiotropic manifestations of this form of dystonia-deafness syndrome based on diverse developmental functions of beta-actin with different temporal susceptibility and compensation by other forms of actin.

## Case presentation

### Clinical symptoms and findings, preoperative diagnostics, and treatment

Daughter of unrelated, healthy parents was born full-term after normal pregnancy with birth weight 2950 g (10th percentile), length 46 cm (2.5th percentile), and head circumference 34 cm (25th percentile). In the neonatal period, parents observed hypotonia, lack of eye contact, weak sucking reflex, and swallowing difficulties with vomiting. From early childhood, she periodically needed enemas due to constipation. Profound hearing loss was diagnosed bilaterally at age 2.5 years and treated with cochlear implants at ages 3 (left) and 12 (right). Psychomotor development was slightly delayed (Table 1). Except for reduced concentration/learning difficulties and hyperactivity, cognitive skills and psychosocial behavior appeared normal until about age 10, and thereafter stagnated.

**Table 1:** Clinical symptoms/development by body region and age, and supplementary diagnostics (except brain imaging)

Body region	Symptom	Age (years)
CNS		
Sensory-motor	Hypotonic, weak sucking reflex, torticollis	Neonatal
	Delayed early motor development––(independent sitting at 11 months, walking at 21 months)	Infancy
	Dystonia	
	Lower limbs (gait dystonia)	12–13
	Upper limbs (jerky action tremor)	14
	Axial (trunk opistotonus, retro- and torticollis, tongue protrusion, jaw opening, face grimacing)	15–16
Cognitive/emotional	Delayed eye contact	Infancy
	Spoken language with few-word sentences, reduced vocabulary. Sign language good	Childhood
	“ADHD”––diagnosis	6–7
	Learning difficulties. Reduced social skills	9–10
	Anxiety, rage outbreaks, hyperimpulsivity	18–19
Auditory system	Bilateral deafness	Infancy
Cranio-facial	Dysmorphic facial features coarser with age Hypertelorism/broad nasal root/flat malar region in infancy, changing to prominent/long nose with thick nares and high nasal root. Mild ptosis bilaterally Dysplastic, simple ears. High palate	Progression with age
Gastro-intestinal	Esophageal reflux/vomiting. Constipation, enemas	Infancy
Musculo-skeletal	Contractures PIP-joint dig. III-V ri. hand + dig.IV-V le	12–13
	Anteverted scapulae, thoracic kyphosis	14
Supplementary diagnostics		
Cranium	No abnormalities on CT or MRI	
Thorax	X-ray, ECG, echocardiography: normal	
Abdomen	Ultrasound, CT: distended duodenum, dilated colon, constipation	
Urinary tract	Ultrasound, including the kidneys: normal	

**Fig. 1:**
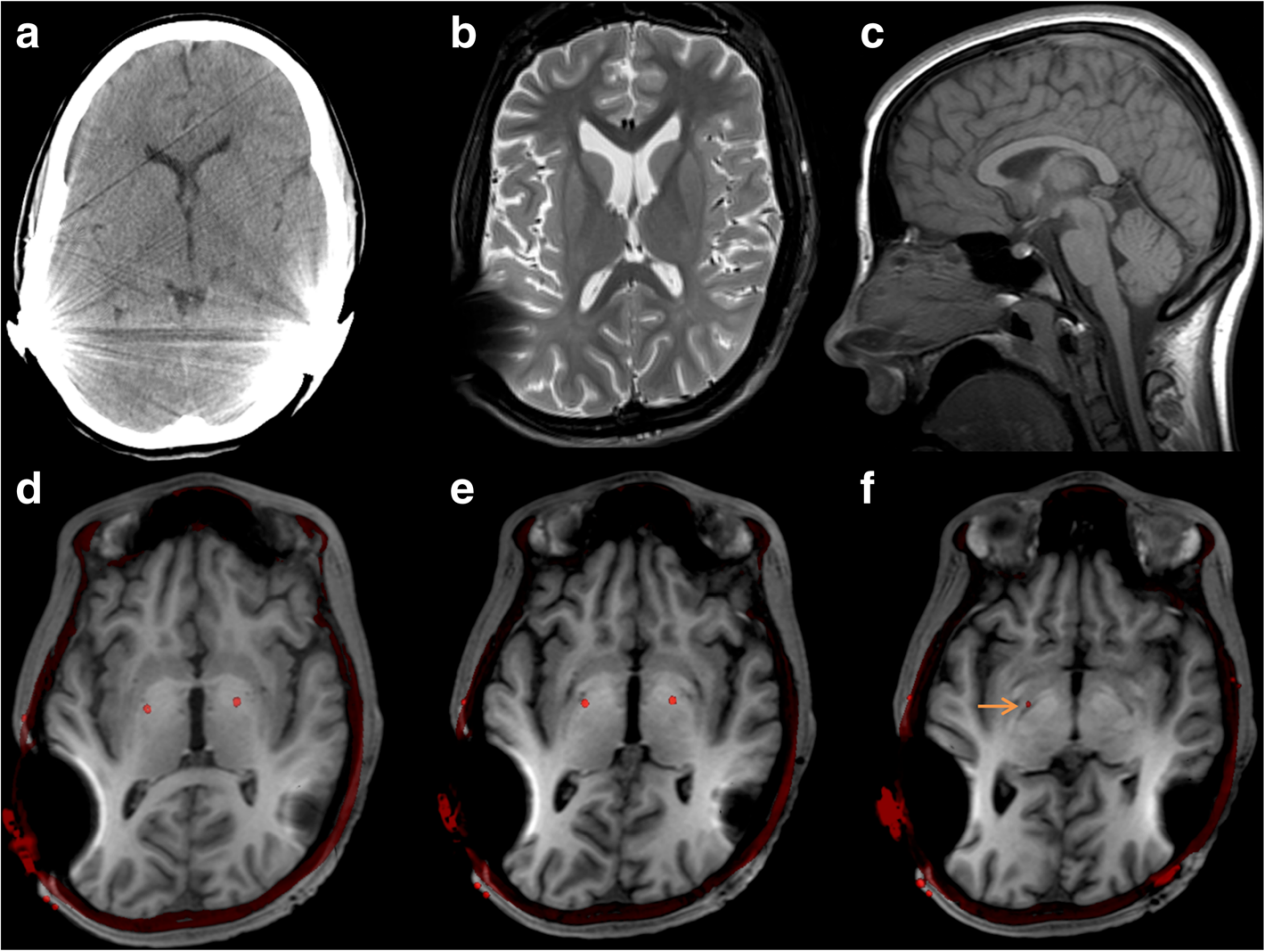
Brain CT (2009) and preoperative brain MRI (2013), fused with postoperative CT. Preoperative MRI (**b**–**f**) did not show evidence of cortical developmental malformations, corpus callosum defects, or any other inborn brain structure anomalies. The maturation of the brain including myelination is normal. Comparison of an axial CT from 2009 (**a**) and MRI T2 from 2013 (**b**) shows enlargement of the lateral ventricles, indicating some supratentorial volume loss over time. A mid-sagittal T1 (2D TSE) image from 2011 (**c**) shows normal configuration and no certain volume loss of the brain stem or cerebellum, and the preoperative MRI series (2013) did not disclose convincing signs of infratentorial volume loss (not shown due to more distorted images). Images **d**–**f** show preoperative T1-3D MPRAGE* (2013) fused with postoperative CT images (2015), showing the position of the DBS electrodes visualized as red dots (image fusion performed using software from NordicICE, NordicNeuroLab, Bergen, Norway). Images **d** and **e** show the electrodes in the posterior third and most ventral part of the internal globus pallidum. Image **f** shows the right electrode tip touching upon the optic tract (orange arrow) (*3D T1 MPRAGE was used for fusion with postoperative CT instead of T2 (which better visualizes the basal ganglia), because the cochlea implants inflicted heavy susceptibility artifacts on the T2 images with pronounced focal signal loss, as well as distortion. Also, the CT alignment/co-registration with the large (whole head) field of view 3D T1 MPRAGE series was accurate, whereas the CT alignment with the (reduced field of view) T2 images was not.)

Motor development was considered normal after infancy. She played soccer, rode horses, had normal handwriting, and made nice drawings. Retrospectively, the dystonia probably started at age 12–13, with action dystonia of both legs manifesting as a clumsy, jumping gait. Dystonic arm tremor appeared at age 14, and over the next 2 years axial dystonia ensued, with spasmodic retro- and torticollis, opisthotonus, and dysarthric speech. Cranial dystonia included grimacing, tongue protrusion, and jaw opening. From age 16, she needed a wheelchair, and from age 17 was mainly confined to bed, in a prone position. At age 18, she was hospitalized due to weight loss and dehydration, resulting from severe constipation, reflux esophagitis, and mild swallowing difficulties. Gastroparesis was suspected (Table 1). When evaluated at our tertiary movement disorders center at age 19, she had constant generalized dystonia, with axial predominance, suspected to be secondary to a complex genetic syndrome due to the associated non-cerebral features (Table 1). Sanger sequencing of multiple single genes and chromosomal microarrays including aCGH and SNP arrays had been performed previously without revealing the molecular diagnosis. Brain MRI showed grossly preserved brain structure, but some lateral ventricle enlargement compared to a CT scan performed 4 years earlier (Fig. 1a–c). Spinal MRI was normal. Severe constipation necessitated oil enemas every other day. Rectal biopsy excluded agangliosis. She weighed 37 kg, which did not increase preoperatively even with full parenteral nutrition, and suffered from repeated life-threatening infections (aspiration pneumonia, upper urinary tract).

Therefore, we implanted a neurostimulation device (Medtronic® Lead Model 3389, Impulse Generator Activa PC) targeting the postero-ventral internal pallidum bilaterally, as described previously [11], in a 1-day procedure (November 6, 2013) under general anesthesia (Fig. 1d–f). Preoperative MRI was obtained the day before, after surgical removal of the cochlear implant magnets, under general anesthesia. As a high-risk patient for stimulator-related infection, she received prophylactic antibiotics from start of procedure until all sutures were removed. From 2 months preoperatively to date, she has received clonazepam 0.75–2 mg q.i.d., mirtazepin or venlafaxine, and from 1.5 years post-surgery levodopa 200–300 mg/day.

After 1 day of stimulation, she could sit relaxed in a chair and lie comfortably in the supine position, and after a month, she could walk with support. Dystonia severity and disability, as assessed by the Burke-Fahn-Marsden Dystonia Rating Scale [12] motor and disability scores, improved from 87/25 points preoperatively, to 21/13 at 2.5 months, 21/11 at 1 year, 25/12 at 2 years, 26/14 at 3 years, and 30/14 at 4 years postoperatively. Stimulation parameters at 4 years are monopolar stimulation, pulse width 90 μs and frequency 150 Hz bilaterally, current left 4.6 mA (cathodes 1 + 2) and right 4.3 mA (cathodes 8 + 9). She has no dystonia at rest, but intermittent activation-induced dystonia in the neck, tongue, right arm, and left leg. Can sit with her head in the midline over time, perform fine motor tasks with both hands, and walk supported with a broad-based gait, with reduced postural stability and a stooped posture (typical for BWCFF). Some slowness and reduced amplitude of repetitive movements may represent mild signs of parkinsonism. A 2017 cognitive evaluation showed mild impairment. Her behavior and emotional responses fluctuate from calmness, concentration, and joy to increased impulsivity, rage outbreaks, and anxiety. She attends school and enjoys physical activities, such as trampoline jumping, swimming, and tandem cycling, and lives with a team of assistants day and night, maintaining close contact with her family.

### Genetic findings

Whole exome sequencing (WES) was performed at the Baylor-Hopkins Center for Mendelian Genomics at Baylor College of Medicine (BCM; Houston, Texas). The WES method, alignment, and variant annotations used at BCM-Human Genome Sequencing Center have been described previously [13]. A heterozygous missense variant, NM_001101.3(ACTB):c.[547C>T];[=], in exon 4 of *ACTB*, leading to the amino acid change p.Arg183Trp was identified. To date, this variant has been reported as disease-causing in six patients [5–9] and is associated with BWCFF type 1 (BRWS1, OMIM#243310) [10].

The suspected disease-causing *ACTB* variant was confirmed by Sanger sequencing performed on genomic DNA from peripheral blood. Primers for Sanger sequencing were designed with Primer3 software and sequenced on an ABI 3730 sequencer (Applied Biosystems, Life Technologies). Sequence data were analyzed with SeqScape v.2.7 (Life Technologies) and 4Peaks. Only a sample from the mother was available for segregation analysis with Sanger sequencing. The mother did not carry the *ACTB* variant. The father was not tested.

### Brain nuclear imaging

Between December 2016 and April 2017, striatal dopaminergic transmission was imaged presynaptically using ^123^I-ioflupane single-photon emission computed tomography (dopamine transporter (DaT)-SPECT) and postsynaptically using ^123^I-epidepride-SPECT, in which the ligand binds with high specificity to dopamine type 2-like receptors in the striatum [14]. Brain glucose uptake was evaluated with ^18^Fluoro-Deoxy-Glucose (FDG)–PET. European Association for Nuclear Medicine guideline compatible protocols were used for each investigation. Tracer uptake was assessed visually and compared with normal databases using commercial software. Results of the quantitative comparisons are shown in Table 2.

**Table 2:** Striatal uptake on radiotracer imaging in our patient compared with mean uptake in normal databases

Brain radiotracer modality	Putamen				Caudate nucleus			
	Right		Left		Right		Left	
	%	SD	%	SD	%	SD	%	SD
DAT-SPECT	72	− 2.6	74	− 2.3	80	− 1.7	82	− 1.5
Epidepride-SPECT	65	− 4.9	76	− 3.4	81	− 2.7	86	− 1.9
FDG-PET		− 4.2		− 3.6		− 3.2		− 3.7

Numbers represent percent of uptake (%) and standard deviation (SD) compared with mean uptake in normal databases for the three radiotracers. DAT (dopamine transporter)-SPECT (^123^I-ioflupane single-photon emission computed tomography) uptake was compared with a commercial database (DaTQuant, GE) that included an extrapolation for age. SPECT with ^123^I-epidepride (which binds to dopamine type 2-receptors) was compared with a non-age-matched database (older patient group, because this modality is very rarely used in the patient’s age group; local data, Hermes BRASS). ^18^Fluoro-Deoxy-Glucose (FDG)–PET was compared with an age-matched database (local data, PMOD PNEURO)

**Fig. 2:**
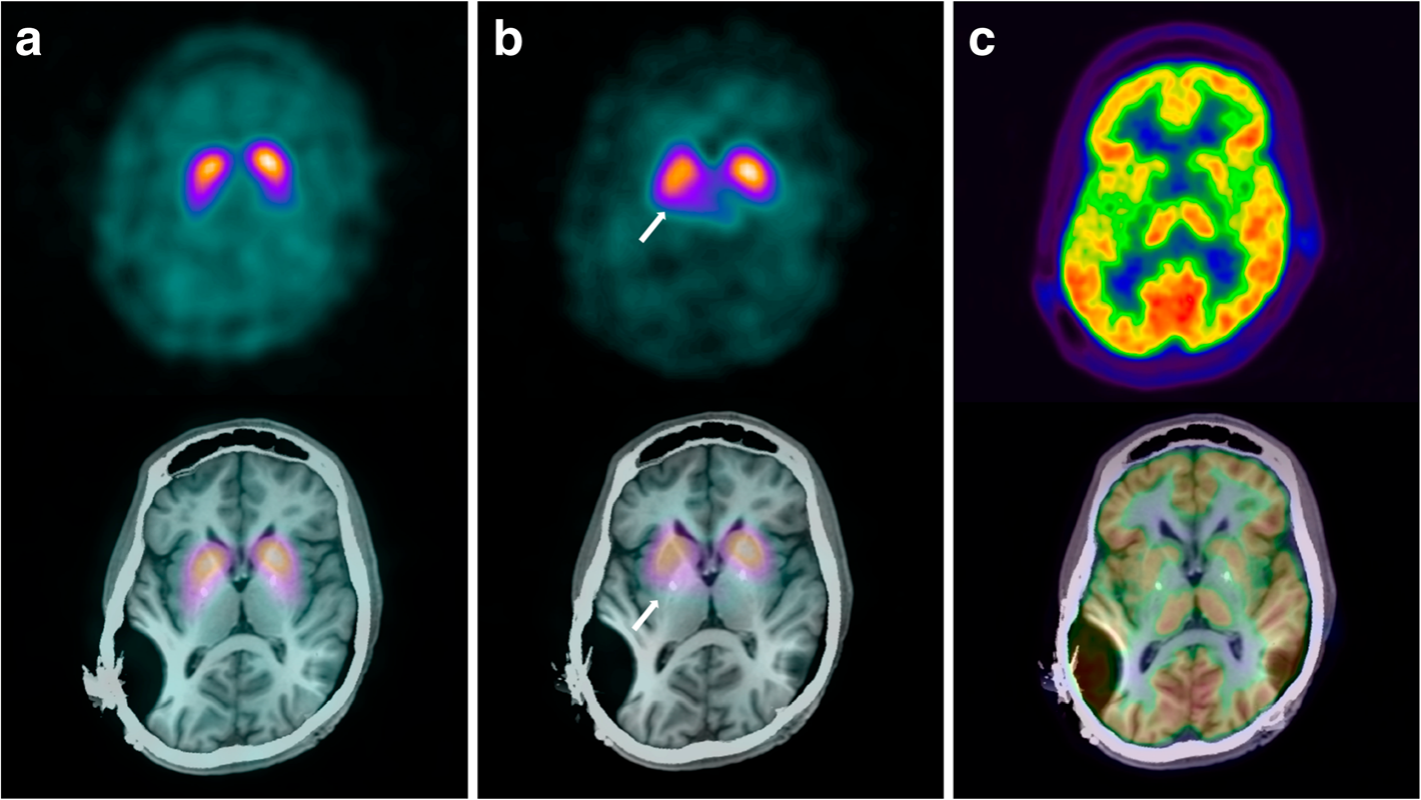
Brain radiotracer imaging of pre- and postsynaptic striatal dopamine transmission and glucose uptake. Upper images show emissions only, and lower images are fused with MRI (T1 series, 2013). **a** ^123^I-ioflupane SPECT-images, with the ligand binding to the dopamine transporter-protein (DaT) of the striatal terminals of the substantia nigra dopamine producing cells (presynaptic). Visual assessment and comparison with a normal database shows reduced binding at around two standard deviations from the database mean, and more pronounced reduction posteriorly in the putamen. **b** ^123^I-epidepride-SPECT images, with the ligand binding to dopamine type 2-receptors (postsynaptic), show significantly reduced binding in the putamen bilaterally compared to non-age-matched (older) normal database mean and less pronounced reduction in the caudate nuclei. Arrows indicate right putamen. **c** ^18^Fluoro-deoxy-glucose PET-images, which reflect the glucose uptake of viable brain cells. Uptake in both striata is lower than in the thalami and the majority of the cortex, compared with age-matched normal images, in keeping with generally reduced striatal uptake

The DAT uptake values are reduced by around two standard deviations (SD) from the mean, and visually the uptake appears lowest posteriorly in the putamen (Fig. 2a). Epidepride-SPECT showed significantly reduced dopamine type 2-receptor binding in the putamen bilaterally and to a lesser extent in the caudate nucleus (Fig. 2b). FDG-PET showed reduced glucose uptake in the entire striatum bilaterally (Fig. 2c).

Metal artifact from cochlear implants in the parietal regions and DBS electrodes may have affected SPECT imaging by scatter and absorption reducing emission, although the effect would be expected to be similar for both DAT and epidepride tracers and relatively small. The metal artifact effect on FDG-PET is expected to result in a small overestimation of uptake on the attenuation corrected images and unlikely to cause the reduction in tracer uptake seen in the entire striatum.

## Discussion

We have presented a now 23-year-old woman with dystonia-deafness syndrome and mild developmental defects compatible with a BWCFF syndrome, caused by the *ACTB* p.Arg183Trp variant. This specific variant has been reported in only six previous patients, who all had similar phenotypes, with childhood-onset sensorineural deafness and adolescent-onset dystonia [5–9]. In our patient, adolescent-onset action dystonia in the extremities progressed axially over a few years to a severe generalized and life-threatening dystonia. Deep brain stimulation of the internal pallidum bilaterally at age 19 was feasible despite previous bilateral cochlear implants and had an immediate beneficial effect that is largely sustained after 4-year follow-up.

Brain imaging data show that in the striatum, and particularly in the putamen, there is both reduced dopamine type 2 (D2)-receptor binding and reduced glucose uptake, indicating reduced function or degeneration of striatal/putaminal neurons, including those that harbor D2-receptors. Also, there is indication of a mild loss of dopaminergic terminals. These unprecedented imaging data corroborate in vivo the post-mortem neuropathological findings of the originally reported monozygotic twin brothers in whom the *ACTB* p.Arg183Trp variant was discovered [5, 6]. Both twins died in their early twenties of aspiration pneumonia secondary to adolescent-onset, severe generalized dystonia. Abundant eosinophilic spherical structures in the striatum, consistent with degenerating neurons and processes, were strongly actin- and actin-depolymerizing factor (ADF)/cofilin-positive, suggesting a defect in actin turnover [5]. Additional actin- and ADF/cofilin-immunoreactivity was noted in the globus pallidus and substantia nigra. Through a systematic search for sequence variants in *ACTB* and *ACTG1*, the p.Arg183Trp variant was found, and lymphoblast cell line studies suggested altered actin cytoskeletal architecture and function, possibly by reducing depolymerization dynamics [6]. In vitro study of p.Arg183Trp ACTB demonstrated a gain-of-function effect: slower actin filament growth, higher ATP-hydrolysis, and faster depolymerization with impaired formation of long, stable filaments [15].

The identical variant was recently described in a mother and daughter [7] with early-onset sensorineural deafness and hand dystonia in late adolescence. Progression to generalized dystonia was rapid in the daughter and slow in the mother. Both responded well to pallidal DBS (at ages 21 and 42, follow-up 12 and 4 months, respectively). The fifth patient was a boy with infant-onset sensorineural hearing loss, mild intellectual disability, and adolescent-onset dystonia with rapid generalization [8]. Left motor cortex stimulation had limited benefit, a status dystonicus ensued, and he died despite medically induced coma. Finally, the same variant was recently identified by WES in a 39-year-old man with severe hearing loss diagnosed at age 3 and reported onset of dystonia at age 24 with retro-torticollis, progressing to generalized dystonia [9], treated from age 29 with good benefit from pallidal DBS [16].

Thus, of the seven patients reported, only the four who received pallidal stimulation have survived. The p.Arg183Trp carriers published were all symptomatic*.* Except for the Dutch family [7], the variant seems to have occurred de novo*,* also in our patient, since both parents are healthy.

The beta-actin protein (ACTB) is an important component of the cytoskeleton and is expressed by all cells in the body along with several other forms of actin [17, 18]. The range of affected organs and symptoms underscores the pleiotropy of *ACTB* disease-causing variants. Midline facial dysmorphogenesis is typical, manifests from a very early age and is likely to be congenital, potentially related to migratory disturbances in cranial neural crest cells that establish facial skeletal structures. Indeed, mouse fibroblasts lacking beta-actin exhibit severe migratory deficiency in vitro and neural crest cells exhibit migratory arrest and subsequent cell death in beta-actin knockout mice [19, 20]. Interestingly, lack of beta-actin in mouse fibroblasts, embryonic stem cells, and embryos leads to compensatory upregulation of gamma- and alpha-actin expression [20]. Such cross-isoform compensation, together with the heterozygosity of the *ACTB* p.Arg183Trp variant, could contribute to the mildness of the cerebrofronto-facial phenotype.

One of the most penetrant phenotypes is early-onset deafness [10]. Although the underlying cause of deafness in *ACTB* patients is unclear, animal studies have demonstrated a critical role of beta-actin in the maintenance of hair cell stereocilia function, with progressive hearing loss starting at 6 weeks of age in *ACTB* knockout mice [21].

Several sequence variants of *ACTB*, as well as of *ACTG1*, severely perturb brain development [10]. By contrast, the *ACTB* p.Arg183Trp variant of our patient (and the six other patients reported to date) is associated with a milder neurodevelopmental deficit, with preserved macroscopic brain structure and only slight delay of initial psychomotor development, indicating no catastrophic or debilitating defects in the main prenatal and early postnatal phases of neurogenesis, axon outgrowth or synaptogenesis.

Intriguingly, overt striatal symptoms including dystonia appear to arise first around puberty in both females and males. Puberty marks a period of substantial changes in striatal volume, synaptogenesis and dopaminergic function, related among other things to adolescent-specific behavioral features related to risk-taking and reward perception [22–27]. Beta-actin is a pivotal molecule in a variety of pre- and postsynaptic processes, and its dysfunction (unless compensated for by other forms of actin) would be expected to impact on synaptic signaling, plasticity, and functions such as neurotrophin uptake [28]. All such effects could compromise striatal function. It is not unlikely that increasing demands placed on striatal connections by pubertal modulation could predispose to deleterious sequelae, including synapse regression, and cell death, when *ACTB* is mutated.

Thus, we propose that the dystonia-deafness syndrome caused by *ACTB* p.Arg183Trp heterozygosity can be explained by specific functions of beta-actin that are compromised in different cell types and cellular functions at different developmental stages. In sequence, these would be neural crest migration and proliferation (facial dysmorphogenesis), hair cell stereocilia function (infant-onset deafness), and synaptic maintenance in the face of pubertal changes in striatal function (adolescent-onset dystonia). Moreover, we propose that partial compensation by the upregulation of other forms of actin, in particular gamma-actin, modulates the degree of severity and the temporal pattern of susceptibility exhibited by the different phenotypic characters. For example, neural crest function is only mildly affected, the striatum is resistant to dystonic pathology until impacted by puberty-related activity and synaptic changes, whereas hair cells are totally incapacitated. It will be interesting to assess the expression patterns of different actin forms in cellular models derived from patients with this particular *ACTB* variant.

In conclusion, this is the seventh patient reported to date with DDS caused by the *ACTB* p.Arg183Trp variant and is one of four patients who have survived the associated severe, generalized dystonia, due to substantial improvement from pallidal stimulation. Brain imaging of dopaminergic transmission in our patient strongly indicates dysfunction and/or loss of striatal neurons, including those harboring dopamine type 2-receptors. We hypothesize that this particular *ACTB* gain-of-function variant renders striatal neurons vulnerable to cell death as a consequence of changing demands on striatal neurons and connections occurring during adolescence and thereby causes dystonia. *ACTB* sequencing should be included in the work-up of dystonia-deafness syndrome. Deep brain stimulation of the internal pallidum bilaterally should be strongly considered to treat generalized dystonia in *ACTB* p.Arg183Trp carriers.

## Abbreviations

BWCFF: Baraitser-Winter cerebrofronto-facial syndrome
DBS: Deep brain stimulation
DDS: Dystonia-deafness syndrome
PET: Positron emission tomography
SPECT: Single-photon emission computed tomography
WES: Whole exome sequencing
